# Modified compact fluorescent lamps improve light‐induced off‐season floral stimulation in dragon fruit farming

**DOI:** 10.1002/fsn3.2088

**Published:** 2021-03-09

**Authors:** Quang Thach Nguyen, Minh Dung Ngo, Thanh Hung Truong, Duy Chinh Nguyen, Minh Chau Nguyen

**Affiliations:** ^1^ Vietnam National University of Agriculture Ha Noi Vietnam; ^2^ Nguyen Tat Thanh University Ho Chi Minh City Vietnam; ^3^ Institute of Agricultural Sciences for Southern Vietnam Ho Chi Minh City Vietnam; ^4^ Southern Fruit Research Institute Tien Giang Province Vietnam

**Keywords:** compact fluorescent lamp, dragon fruit, floral stimulation, flowering control, photon flux density

## Abstract

Efficient light‐induced floral stimulation plays a key role in energy conservation and maintaining stable productivity during off‐season periods of dragon fruit plants. In this study, we first reported on results of a survey on dragon fruit farmers regarding use of lamps in performing artificially induced flowering process in Vietnam. It was found that the use of incandescent lamp was prevalent in dragon fruit cultivation practices, resulting in heavy electricity consumption, and that low‐power compact fluorescent light (CFL) bulbs were not extensively utilized, possibly due to low floral induction performance of domestic CFL bulbs. Arguing that emission spectra of currently used lamps were not consistent with adsorption spectra of phytochromes, whose transformation is responsible for flowering process of dragon fruit, we then proposed three improved CFL lamps (power capacity of 20 W) having emission spectra focused on red and far‐red regions. New lamp prototypes were tested in 7 field experiments in three different provinces in Vietnam. One improved CFL bulb (treatment 2) performed relatively well in comparison with the incandescent control lamp (60 W) in six out of seven experiments with regard to some growth indicators (e.g., number of floral stems, number of bubs, number of fruits per plant) and fruit yield. Recent success on commercialization of the improved CFL lamp demonstrates the potential of CFL lamps in floral stimulating irradiation of other crops and plants and in alleviating electricity burden in dragon fruit growing areas.

## Graphical abstract

The study first surveyed on dragon fruit farmers on measures to stimulate flowering in commercial dragon fruit farming in three provinces of Vietnam. We then modified emission spectra of Compact Fluorescent Lamps and used them in field experiments. One improved low‐power CFL bulb showed flowering induction capability comparable to the incandescent bulb.

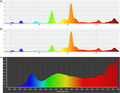

## 1 INTRODUCTION

Vietnam has been one of the major exporters of dragon fruit (pitaya) in the world, possessing about 35 000 ha of total commercial cultivation area in 2015 (Ministry of Agriculture & Rural Development of Vietnam, 2019a). Among regions specialized in agriculture in Vietnam, Binh Thuan Province is the area with the largest dragon fruit farming with 23 200 ha reserved solely for dragon fruit farming (Ministry of Agriculture & Rural Development of Vietnam, 2019b). In 2017, export turnover of twelve spearhead exporting fruits from Vietnam contributed around 2.6 USD billion to the national net exports. Of which, dragon fruit accounted for 44.23%, which is 1.15 USD billion and is expected to grow exponentially in the coming years due to growing interest in the fruit in the global market (Nguyen, 2020; Vietnam General Department of Customs, 2017).

Dragon fruit is a photoperiodic plant that optimally blooms in the geographic condition in which daytime is longer than nighttime (Su, 2005). As such, in Vietnam, main cropping season of dragon fruit spans from March to September in the following year and cultivation in the remaining period of the year should be aided with artificial irradiation during nighttime. Off‐season floral stimulation of dragon fruit is especially important for farmers because Lunar New Year, which is the most important holiday in Vietnamese culture and has greatly increased demand for fruit products, often falls in the end of the off‐season period. This causes the off‐season harvest to be the major income source of dragon fruit farmers and necessitates the use of lamps to stimulate off‐season flowering. Currently, high‐power incandescent lamps with capacity ranging from 75 W to 100 W are widely used to provide irradiation for 4–6 hr per night to dragon fruits in Taiwan (Tran et al., 2015; Yen & Chang, 1997) and Thailand (Saradhuldhat et al., 2009, 2016). Incandescent light bulbs with capacity ranging from 75 W to 100 W are also recommended by Vietnamese Ministry of Agriculture and Rural Development (MARD) to provide around 10 hr of artificial lighting per night (Hoa et al., 2008; Ministry of Agriculture & Rural Development of Vietnam, 2006; Truong, 1999).

To our knowledge, there have been four studies that investigated the effect of irradiation on flowering of dragon fruit plant. The study of Khaimov and Mizrahi (2006) attempted the manipulation of flowering pattern of pitaya (*Hylocereus undatus*) and yellow pitaya (*Selenicereus megalanthus*) via a number of interventions including photoperiodic lighting, shading, flower thinning, and application of growth regulators. It was concluded that even 9 hr of lighting extension after sunset induced no effect on flowering, possibly due to the subtropical climate in the study area (Israel). This explanation was further confirmed by Jiang et al. (2012) where temperature‐sensitive response to night‐breaking fluorescent lighting (28 W) was found in red pitaya cultivated in Taiwan. However, light‐stimulated fruit production during off‐season period was lower than that in the inductive period and it was unclear whether the use of incandescent lamps or fluorescent lamps at higher capacity could improve off‐season pitaya production. Similarly, Saradhuldhat et al. (2009) reported good stimulation effect of 2‐hr night‐break lighting using fluorescent tube on pitaya cultivated in Chanthaburi, Thailand. However, the experiment in this study was of model‐scale and only performed in four replications of four plants. Thus, it is difficult to justify the result on a larger scale. On the other hand, Tran et al. (2015) indicated that the response to artificial lighting might also vary depending on the species, regional cultivar, and flesh color. Most notably, the Vietnam origin white‐flesh pitaya cultivar was unable to flower under 4‐hr night‐breaking irradiation with incandescent bulbs (100 W).

In general, the aforementioned studies was unable to show the flowering response of pitaya to different lighting conditions (e.g., lamp type, power capacity, and emission spectrum) and was conducted in different regions having varying temperature. As a result, obtained results are contradictory and carry little implication when being implemented in regions with different climatic conditions. Driven by such shortcomings and the high electricity consumption in areas with intensive dragon fruit cultivation in Vietnam, this study aims to rationalize a low‐capacity compact fluorescent lighting (CFL) lamp that is specifically designed for controlling off‐season flowering of dragon fruit. We first presented the results on the current measures for controlling off‐season dragon fruit flowering in three typical areas in Vietnam, namely Tien Giang, Tay Ninh, and Binh Thuan Province. Then, some CFL configurations were proposed and analyzed for their light spectra. Lastly, the selected CFL lamps were then used in experiments carried out in these areas to assess their stimulation effectiveness on some growth indicators of dragon fruits of both red‐flesh and white‐flesh varieties. Current results are expected to contribute to alleviate electricity burden for agricultural activities in those areas and aid in further development of CFL lamps specialized for fruit irradiation.

## 2 MATERIALS AND METHODS

### 2.1 Survey of lamp use for off‐season blooming control of dragon fruits

A questionnaire was designed aiming at collecting relevant information on dragon fruit farming and techniques for flowering stimulation from farmers in Vietnam. Some typical categories include type of lamp, irradiation process, irradiation duration, and bulb density per hectare. A total of 100 respondents were selected from the farmer lists prepared by the local authorities in three provinces, including Binh Thuan (50 households), Tien Giang (25 households), and Tay Ninh (25 households) according to probabilistic randomized sampling procedure. We also collected lamp samples that were being used for floral stimulation from local farmers for further analysis.

### 2.2 Lamp spectrum analysis

A PG100N Handheld Spectral PAR Meter (UPRtek, Miaoli, Taiwan) connected to a computer was used to measure emission spectra and determine photosynthetically active photon flux density (PPFD), photon flux density in the red region (PFD R, 600–700 nm), and photon flux density in the far‐red region (PFD FR, 700–780 nm) of lamps. The testing lamp was positioned 30 cm in front of the instrument sensor in the dark. The procedure was carried out at Laboratory of Agricultural Biotechnology, Nguyen Tat Thanh University, Vietnam.

### 2.3 Field experiments

Based on the survey results and spectrum analysis, we designed new CFL bulbs with spectra focused in the red and far‐red regions. The light bulbs were manufactured by Rang Dong Light Source & Vacuum Flask Joint Stock Company (Vietnam) and were screened before being used in field experiment.

In the field experiment, three experimental CFL bulbs (20 W) selected from the spectra analysis results were used as treatments (denoted from 1 to 3) and one commercial incandescent lamp (60 W) acted as the control bulb (denoted as control). No lighting and domestic CFL lighting were not included as controls due to zero or very low productivity resulted from the adoption of the two options. In addition, Yamada et al. (2008) also maintained that the use of domestic CFL lighting gave longer budding time and worse flowering efficiency.

The subject plants were mature white‐flesh pitaya (*Hylocereus undatus*) and red‐flesh pitaya (*Hylocereus polyrhizus*) that were normally bearing fruits with the age ranging from 5 to 8 years. The experiment was carried out in household fields located in three provinces with largest dragon fruit production in Vietnam, namely Binh Thuan, Tien Giang, and Tay Ninh (Table 1). In each province, one field was selected to perform the experiment. Of the three fields, the field that was located in Binh Thuan province and specialized in cultivating white‐flesh pitaya was experimentally employed for three consecutive harvesting seasons. Meanwhile, the other two fields solely focused on cultivation of red‐flesh pitaya and were treated with experimental light bulbs only for two seasons.

**TABLE 1:** Description of field experiments in the study

Experiment	Pitaya variety	Experiment period	Field location
1	White flesh	Sep. 2013 to Nov. 2013 (first harvesting season)	Ham Minh commune, Ham Thuan Nam district, Binh Thuan province
2	White flesh	Jan. 2014 to Mar. 2014 (second harvesting season)	Ham Minh commune, Ham Thuan Nam district, Binh Thuan province
3	White flesh	Oct. 2014 to Dec. 2014 (third harvesting season)	Ham Minh commune, Ham Thuan Nam district, Binh Thuan province
4	Red flesh	Sep. 2013 to Nov. 2013 (first harvesting season)	Quon Long commune, Cho Gao district, Tien Giang province
5	Red flesh	Oct. 2014 to Dec. 2014 (third harvesting season)	Quon Long commune, Cho Gao district, Tien Giang province
6	Red flesh	Jan. 2014 to Mar. 2014 (second harvesting season)	Gia Loc commune, Trang Bang district, Tay Ninh province
7	Red flesh	Oct. 2014 to Dec. 2014 (third harvesting season)	Gia Loc commune, Trang Bang district, Tay Ninh province

**SCHEME 1:**
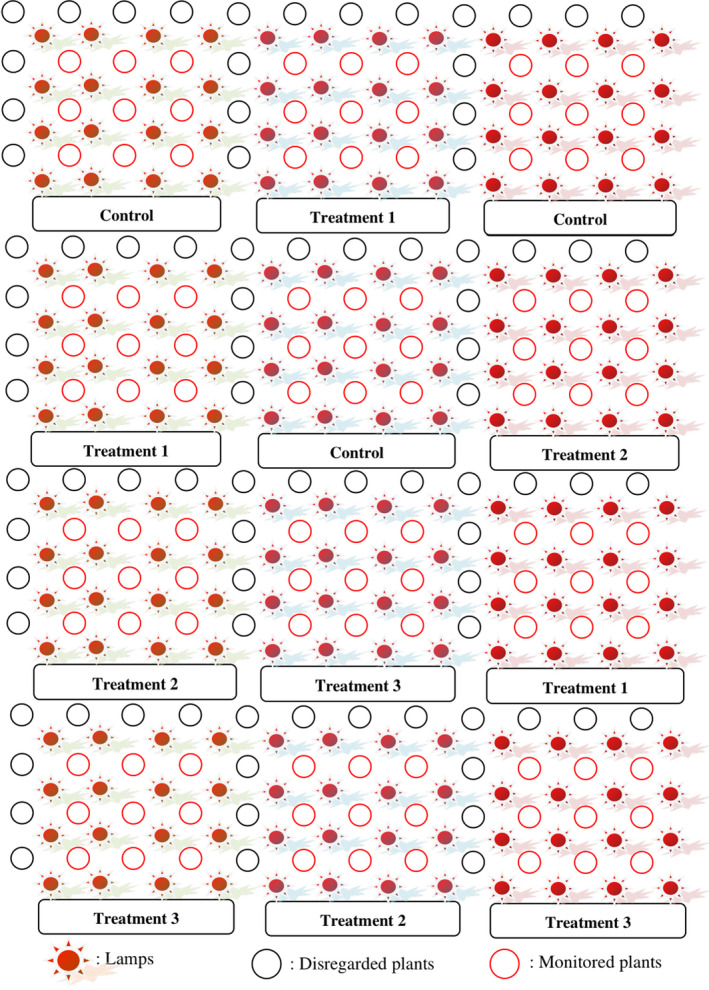
Plant and lamp arrangement in experiment 1

White‐flesh dragon fruit plants were irradiated for 17 consecutive nights with each night involving around 11.5 hr of continuous irradiation starting from 18:30 p.m. to 6:00 a.m. For red‐flesh plants, artificial lighting commenced for 14 consecutive nights and the plants were continuously irradiated for 8 hr starting from 21:00 p.m. to 5:00 a.m. each night. These timings and durations were selected based on current cultivation practices for commercial fruit production during off‐season periods. After stopping irradiation for 7–10 days, pitaya plants began to develop floral buds. Plants blossomed after 25–28 days since artificial lighting had ended. Fruits fully developed and were harvested after 55–60 days following lighting stoppage. Experiments in each field were designed according to Randomized Completely Block Design with four replications for each treatment. Each replication consisted of 16 plants. Of which, only 9 plants were monitored, and the remainder were neglected due to light interference between different lamps (Scheme 1). Watering, fertilization, use of chemicals, and tending procedure followed the guideline recommended by MARD (Ministry of Agriculture & Rural Development of Vietnam, 2006).

### 2.4 Statistical analysis

Excel software was used in producing descriptive statistical analysis. The experiments were calculated according to the method for field survey (Gomez & Gomez, 1984). Average monitoring parameters of each replication between experimental treatments were treated by the method of analysis of variance (ANOVA), then compared with Duncan's test at confidence level *p* ≤ .05 by using SAS software 9.1.

## 3 RESULTS AND DISCUSSION

### 3.1 Current status on bulb use for controlling off‐season flowering of dragon fruit

Preliminary interviewing results on some farmers and daytime measurement in the three provinces that have largest commercial dragon fruit cultivation area in Vietnam (Binh Thuan, Tien Giang, and Tay Ninh province) confirmed that, from September to March of the following year, dragon fruit plants cultivated in these areas were unable to bloom under natural conditions. In fact, this is also the period in which the daytime is shorter than 12 hr. As a result, a majority of farming households adopted artificial irradiation as a measure to stimulate flowering of dragon fruit crops. Prior to 2012, off‐season floral stimulation of dragon fruit often involved incandescent bulbs with the capacity of either 100 W, 75 W, or 60 W to supplement lighting during nighttime. In the years 2013–2014, there was a shift in the use of bulbs for dragon fruit flowering induction toward incandescent bulbs with lower capacity (40 W) and compact fluorescent bulbs (20 W) to save electricity. To gain further insights into farmers' behavior in dragon fruit farming, a typical sample of 100 farming households located in the three regions was surveyed for their use of light bulbs in off‐season harvests. Table 2 showed the summarized survey results under some main indicators.

**TABLE 2:** Number of surveyed dragon fruit farming households categorized by some measurements regarding the use of artificial irradiation for induced flowering of dragon fruit in three typical provinces (survey results as of Jan. 2014)

Location	Tien Giang (red flesh)	Tay Ninh (red flesh)	Binh Thuan (white flesh)
Total no. of surveyed households (*n* = 100)	25	25	50
No. of households divided by type of used lamps			
INC	23	12	46
CFL	1	8	1
INC + CFL	1	5	3
Others	0	0	0
No. of households divided by power capacity of used lamps			
25 W	1	8	2
40 W	1	5	3
60 W	23	12	45
>60 W	0	0	0
No. of households divided by irradiation duration			
<6 hr/night	0	0	0
7 hr/night	0	0	0
8 hr/night	25	23	0
9 hr/night	0	2	10
>9 hr/night	0	0	40
No. of households divided by time at which artificial irradiation starts			
19:00 p.m.	0	0	40
20:00 p.m.	0	2	10
21:00 p.m.	25	23	0
After 21:00 p.m.	0	0	0
No. of households divided by no. of off‐season harvests with artificial irradiation per year			
2 times	0	0	0
3 times	23	25	47
4 times	2	0	3
No. of households divided by lamp use and average yield per plant			
INC and >10 kg/plant	23	14	45
INC and <10 kg/plant	1	3	3
CFL and >10 kg/plant	0	1	0
CFL and <10 kg/plant	1	7	2
No. of households divided by bulb density per hectare			
1,000–1,200 bulbs	20	22	44
1,600–1,800 bulbs	5	3	5
>2,000 bulbs	0	0	1

On the whole, it was showed that all surveyed household relied on artificial lighting to control blossoming of both white and red‐flesh dragon fruits. Incandescent light bulbs with power capacity of 40 W and 60 W were the dominantly used lamps for the floral stimulation while the households that used CFL bulbs (20 W) only accounted for around 10% of the total surveyed respondents. Moreover, no other types of bulbs were recorded for dragon fruit irradiation purpose. Measures aimed to improve energy efficiency of irradiation, such as intermittent lighting were not adopted (Saradhuldhat et al., 2009).

There is a discrepancy in bulb use between Tay Ninh and other two provinces. To be specific, the percentage of households using CFL for dragon fruit irradiation in Tay Ninh was 32%, which was higher than that recorded in both Tien Giang and Binh Thuan (4%). Tay Ninh is also the province in which many households adopted the combinational irradiation with both incandescent and CFL light bulbs. In addition, there were 5 out of 25 surveyed households in Tay Ninh reporting the use of incandescent bulbs with lower capacity of 40 W. Higher adoption of CFL in Tay Ninh could be explained by the recently emerged inclination to commercially grow dragon fruit in the province, as opposed to the other two regions where cultivation traditions have already been established; thus, the local farmers are hesitant to endorse new, improved growing techniques.

Another notable feature is that growers in Binh Thuan province started their artificial lighting earlier than those in Tay Ninh and Tien Giang. Specifically, the latest time in Binh Thuan in which irradiation started was 19:00 p.m. meanwhile 100% of farmers in Tien Giang and 92% of surveyed Tay Ninh growers commenced their lighting 2 hr later, at 21:00 p.m. Two households who were able to begin early irradiation in Tay Ninh also reported the use of CFL bulbs, which are less energy consuming than incandescent counterparts, in their fields. This is reportedly due to delayed provision of electricity source reserved for agricultural activities and possibly reflects the inadequacy between grid capacity and production readiness of the electricity supplier and the recent, rising energy needs for dragon fruit cultivation in these areas.

Regarding lamp density, most growers allocated around 1,000–1,200 bulbs per hectare of cultivation. Higher distribution densities were only implemented in the harvest season from January to March where dragon fruits face difficulties in blooming. Farmers also reported that, during this time period, white‐flesh dragon fruit plants were less susceptible to light‐induced floral stimulation than red‐flesh plants and in some cases in Binh Thuan, a denser distribution of more than 2,000 bulbs per hectare was required. With respect to lamp use specific to dragon fruit variety, there was no indication suggesting light bulb preference against certain variety. In Binh Thuan, 45 out of 50 surveyed growers specializing in white‐flesh variety mainly utilized incandescent lamps for floral stimulation. Of which, incandescent bulbs with capacity of 60 W and 40 W accounted for nearly 90% and 6%, respectively. In two areas specialized in cultivating red‐flesh dragon fruit, the percentages are also similar in which only 4% of households adopted CFL lamps.

Overall, from the survey results, it is indicated that efficient control of off‐season blossoming is an important concern in commercial dragon fruit farming and that possible improvements should be called for in efforts to conserve energy for such processes. Currently, all dragon fruit farmers adopted artificial irradiation as the main measure for controlling off‐season flowering and ruled out other stimulation methods such as chemical intervention and foliar fertilization. Moreover, the irradiation process was carried out based on farmers' experience, thus lacking considerations on standardizing critical aspects including bulb type, height of bulb hanging, bulb density, irradiation duration, and implementation of intermittent lighting.

### 3.2 Spectrum analysis of currently used lamps for controlling off‐season flowering of dragon fruit

**FIGURE 1:**
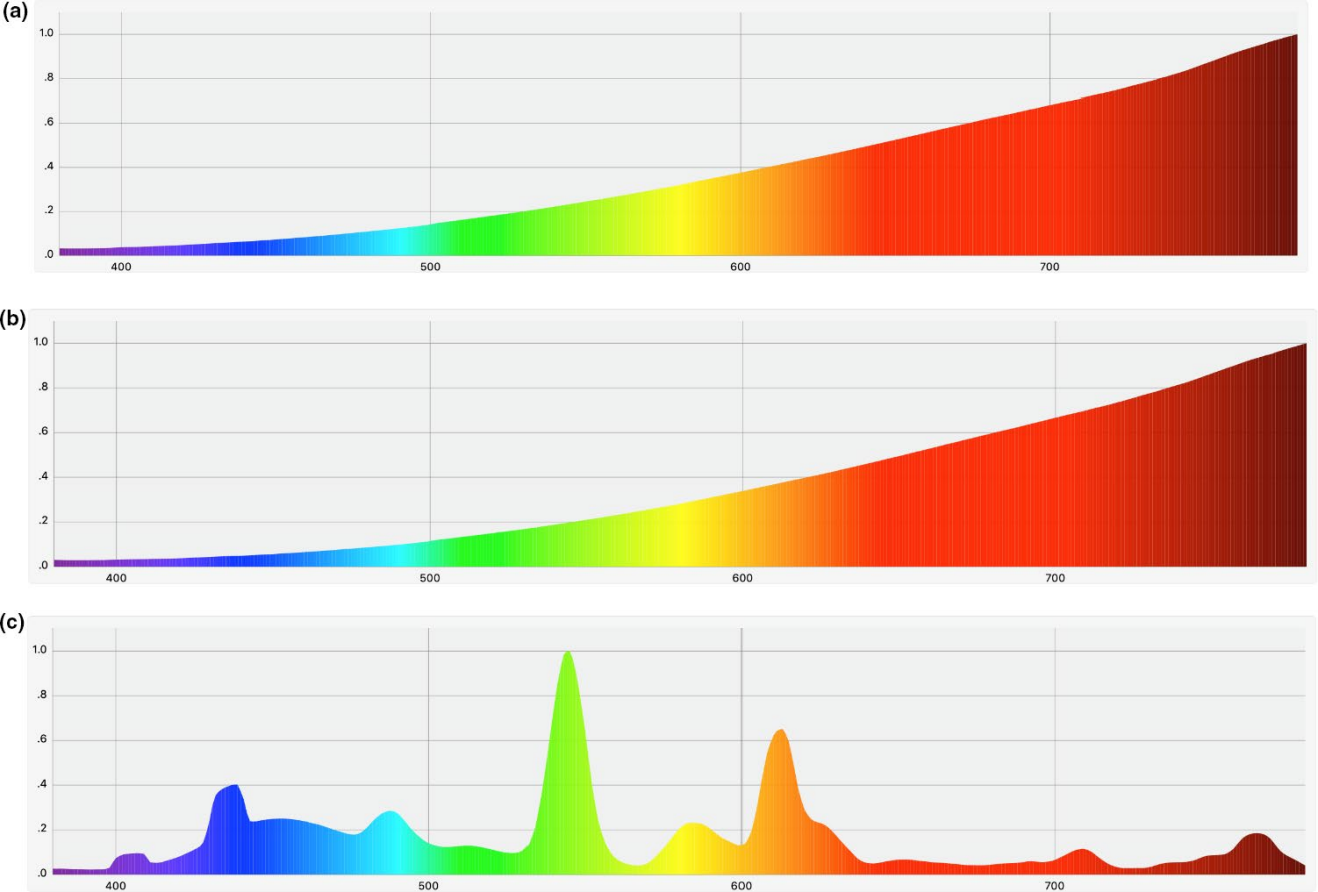
Emission spectra of incandescent lamps with capacity of 60 W (a) and 40 W (b) and of CFL lamps (c) with capacity of 20 W that were currently in use

We collected three types of lamps commonly used by dragon fruit farmers (60 W incandescent, 40 W incandescent lamp, and 20 W domestic CFL lamp) and measured their emission spectra. The results are shown as in Figure 1.

**FIGURE 2:**
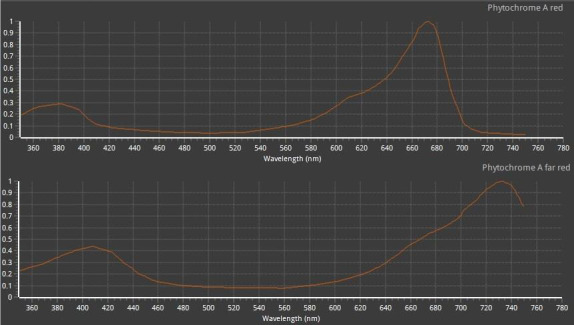
Absorption spectra of two forms of phytochrome

Examination of the spectrum of the three lamps shows that two incandescent lamps exhibited great intensity in the far‐red and red region of their emission spectra, which coincides with the absorption spectrum of two main forms of phytochrome, namely P_660_ and P_730_ (Figure 2). Both of them are mutually transformable under appropriate irradiation and dark conditions and the ratio between the two determines blossoming state of the plant (Taiz & Zeiger, 1991). The consistency between intensities of far‐red and red regions of the incandescent lamp emission spectra and maximum absorption wavelength in P_660_ and P_730_ absorption spectra also sides with the widespread use of incandescent lamps with the capacity ranging from 60 to 100 W for dragon fruit flowering control in Taiwan and in Thailand (Saradhuldhat et al., 2009; Yen & Chang, 1997). However, the spectral regions that are not consistent with absorption spectra of phytochromes, including yellow, green, blue, and dark red areas, of incandescent lamps also exhibited high intensities. Most notably, the dark red region with the wavelength >730 nm, which is close to infrared light, displayed the greatest intensity compared with other regions, indicating possibly redundant heat and energy waste when the bulbs are used for floral stimulation.

The spectrum of the 20 W domestic CFL lamp (Figure 1c) displayed high intensity in the green and yellow light, which is engineered to suit the conventional lighting for human eye. The CFL lamp also has fair intensity in the far‐red and red region but is comparatively lower than that in the absorption spectra. To further highlight the difference in red and far‐red intensities between the two types of lamps, we measured the photon density of the two emission regions. The results are summarized in Table 3.

**TABLE 3:** Photosynthetically active photon flux density (PPFD), photon flux density in the red region (PFD R, 600–700 nm) and photon flux density in the far‐red region (PFD FR, 700–780 nm) of incandescent, and compact fluorescent lamps (µmol m^−2^ s^−1^) that were currently used for floral stimulation of dragon fruit

Type of lamps	PPFD (µmol m^−2^ s^−1^)	PFD R (µmol m^−2^ s^−1^)	PFD FR (µmol m^−2^ s^−1^)	Total PFD (from red (R) to far‐red (FR)) (µmol m^−2^ s^−1^)
INC 40 W	22.4	15.6	23.1	38.7
INC 60 W	44.5	29.6	41.7	71.3
Domestic CFL 20 W	21.4	6.37	3.32	9.69

In terms of PPFD, the 60 W incandescent type exhibited the highest value, at 44.5 µmol m^−2^ s^−1^, which is twofold higher than that of 40 W counterpart and of the 20 W CFL. PPFD of the 40 W incandescent and 20 W CFL bulb fell within a narrow range from 21.4 to 22.4 µmol m^−2^ s^−1^, suggesting that they have similar photosynthetic effects. However, analysis results of photon density in the red and far‐red regions revealed that two incandescent lamps had different capacities in terms of controlling phytochrome transformation. To be specific, the 60 W incandescent lamp gave the highest total PFD (71.3 µmol m^−2^ s^−1^), followed by the 40 W incandescent lamp (38.0 µmol m^−2^ s^−1^). The 20 W CFL lamps usually have total PFD at the lowest value (9.69 µmol m^−2^ s^−1^).

Clearly, currently used lamps all had emission spectra focused on red and far‐red regions. Of which, two incandescent lamps showed that the density of the far‐red and red regions were proportional to the bulb capacity and the CFL lamp usually have much lower red and far‐red photon density than incandescent lamps. As a result, the use of domestic CFL lamps might induce weaker floral stimulation effect on dragon fruit cactus than incandescent counterparts, even at similar power capacities. This is in part illustrated by the previous survey results where only 9% of surveyed farmers with CFL irradiation reported the productivity of higher than 10 kg per plant, as opposed to 92% of growers using incandescent lamps reported the same yield. Current results suggest possible improvements in stimulation efficiency could be achieved by manipulating spectral intensity of CFL lamps toward increased far‐red and far‐red photon density.

### 3.3 Proposed CFL lamps and field experiment results on their effects on some growth indicators of dragon fruits

**FIGURE 3:**
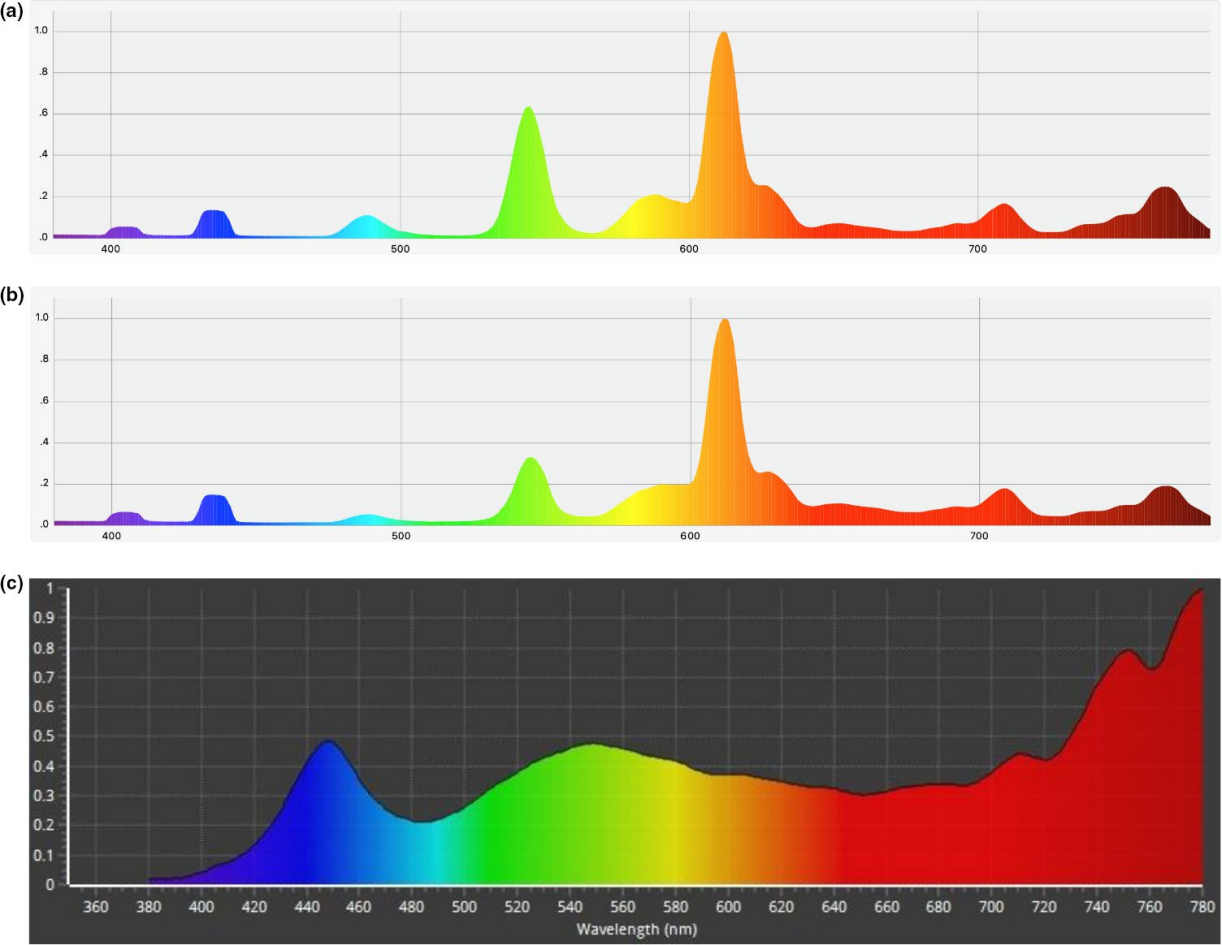
Emission spectra of three improved incandescent lamps which were used as treatment 1 (a), treatment 2 (b), and treatment 3 (c). All bulbs had the power capacity of 20 W and voltage of 220 V

We obtained various improved CFL specimen lamps from Rang Dong Light Source & Vacuum Flask Joint Stock Company, one of leading manufacturers of light bulbs in Vietnam. All bulbs had the power capacity of 20 W and were specifically designed with increased intensity in red and far‐red regions and reduced green and yellow intensity in their emission spectra. After measuring their spectra, we selected three CFL bulbs with highest total PFD and used them in the following field experiment. The spectra and analysis results are shown as in Figure 3 and Table 4.

**TABLE 4:** Photosynthetically active photon flux density (PPFD), photon flux density in the red region (PFD R, 600–700 nm), and photon flux density in the far‐red region (PFD FR, 700–780 nm) of CFL lamps (µmol m^−2^ s^−1^) that were used in field experiments

Treatment CFL bulb	PPFD (µmol m^−2^ s^−1^)	PFD R (µmol m^−2^ s^−1^)	PFD FR (µmol m^−2^ s^−1^)	Total PFD (from red (R) to far red (FR)) (µmol m^−2^ s^−1^)
Treatment 1	19.2	13.8	4.73	18.53
Treatment 2	18.4	14.7	5.58	20.28
Treatment 3	23.2	7.69	10.6	18.29

Three improved CFL bulbs were used in field experiments conducted in three main fields located in three typical provinces with commercial dragon fruit farming activities. The experiments spanned across multiple harvest seasons of the year from 2013–2014 and were conducted on the two varieties of dragon fruit. Incandescent lamp with the capacity of 60 W was used as the control. The results are presented in Table 5.

**TABLE 5:** Effects of lighting with different types of improved CFL lamps on growth indicators and productivity of white‐ and red‐flesh varieties of dragon fruit grown in Binh Thuan, Tien Giang, and Tay Ninh province

Indicators	No. of stems per plant	No. of floral stems per plant	No. of buds per plant	No. of fruits per plant	Fruit weight (g)	Yield per plant (kg/plant)
Experiment 1 (Binh Thuan, Sep. 2013–Nov. 2013, 1st harvest)						
Control	120.1	19.3a	25.6a	21.3a	500	12.7a
Treatment 1	122.5	15.1b	18.4c	16.7b	480	8.90b
Treatment 2	121.3	18.9a	25.4a	21.6a	500	12.6a
Treatment 3	119.9	12.5b	16.5c	16.3b	480	7.90c
CV (%)	3.64	11.5	1.45	10.39	17.0	2.09
LSD_0.05_	ns	3.51	3.20	3.74	ns	0.9
Experiment 2 (Binh Thuan, Jan. 2014–Mar. 2014, 2nd harvest)						
Control	119.0	1.1b	2.0b	2.0b	670.2b	1.3b
Treatment 1	119.4	1.3b	2.4b	2.4b	710.7a	1.5b
Treatment 2	121.2	3.4a	6.7a	6.7a	579.1c	4.1a
Treatment 3	120.1	3.0a	6.1a	6.1a	590.6c	3.8a
CV (%)	3.0	16.2	30.2	10.2	17.9	15.17
LSD_0.05_	ns					
LSD_0.01_		1.1	3.9	3.9	39.5	3.5
Experiment 3 (Binh Thuan, Oct. 2014–Dec. 2014, 3rd harvest)						
Control	124.3	30.9a	60.4a	29.4a	457.9	13.8a
Treatment 1	118.6	23.5b	42.3b	22.0b	455.9	10.1b
Treatment 2	121.9	30.0a	59.4a	29.3a	441.8	13.0a
Treatment 3	121.2	22.0b	40.7b	22.7b	436.8	9.9b
CV (%)	3.75	10.7	8.6	1.36	2.35	3.75
LSD_0.05_	ns	5.7				
LSD_0.01_			8.7	3.2	ns	2.8
Experiment 4 (Tien Giang, Sep. 2013–Nov. 2013, 1st harvest)						
Control	156.7	41.4a	66.7a	39.5a	385.6	15.1a
Treatment 1	153.7	31.3b	48.6b	35.1b	375.2	13.2b
Treatment 2	165.8	30.4b	47.8b	34.9b	371.1	12.9b
Treatment 3	160.8	27.7b	42.4b	36.9b	376.2	13.3b
CV (%)	6.2	12.3	16.9	17.8	12.06	11.98
LSD_0.01_	ns	8.0	17.4	2.5	ns	1.78
Experiment 5 (Tien Giang, Oct. 2014–Dec. 2014, 3rd harvest)						
Control	165.8	14.2a	20.4a	20.4a	490.9	10.8a
Treatment 1	153.7	4.2b	6.4b	6.4b	725.7	4.9b
Treatment 2	163.6	12.1a	18.7a	18.7a	551.2	10.4a
Treatment 3	160.8	6.3b	8.7b	8.7b	640.6	5.4b
CV (%)	5.47	27.29	28.24	28.24	14.78	21.34
LSD_0.01_	ns	4.42	6.67	5.2	ns	4.8
Experiment 6 (Tay Ninh, Jan. 2014–Mar. 2014, 2nd harvest)						
Control	148.8	2.6b	4.3c	4.3c	730.9	3.2c
Treatment 1	129.5	6.8ab	9.9ab	9.9ab	643.6	6.5ab
Treatment 2	132.3	9.1a	14.3a	14.3a	580.2	8.3a
Treatment 3	133.3	6.5ab	9.0bc	9.0bc	649.5	5.8b
CV (%)	10.2	23.2	18.1	18.1	17.41	21.09
LSD_0.01_	ns	4.4	5.1	5.1	ns	2.2
Experiment 7 (Tay Ninh, Oct. 2014–Dec. 2014, 3rd harvest)						
Control	148.8	22.4a	39.1a	29.1a	458.1	13.8a
Treatment 1	125.9	16.5b	24.3b	21.5b	455.9	9.9b
Treatment 2	133.3	21.0a	37.7a	28.9a	441.8	12.9a
Treatment 3	132.3	14.3b	22.3b	20.9b	436.8	9.3b
CV (%)	9.97	9.9	13.2	24.2	5.06	4.98
LSD_0.01_	ns	3.7	8.1			
LSD_0.05_				7.2	ns	2.82

Note

Means with the same letters within each column are not significantly different at *p* ≤ .05 indicated by Duncan multiple range test.

Plant height, canopy diameter, and the number of stems per plant before and after the experiment 1 were not significantly different (See Table A1 in the Appendix A), confirming that the irradiation treatment was not affected by varying plant characteristics during the experiment.

The number of floral stems and the number of buds per plant are important measures for assessing sufficiency of irradiation supplementation facilitating the transition from vegetative into reproductive stage of plants. All four types of lamps in the field experiment were capable of inducing light‐stimulated flowering, albeit at fluctuating extent. In experiment 1, the number floral stems per plant for plants irradiated with treatment 2 (18.9) is statistically comparable to that of the control (19.3) and is higher than those illuminated by two other CFL lamps (15.1 and 12.5). For the indicator of number of buds per plant, a similar trend was found where the control and the treatment 2 both gave almost equal results (25.4 and 25.6, respectively) and treatments 1 and 3 showed inferior bud‐stimulating efficacy (18.4 and 16.5, respectively). The better flowering of plants treated with the incandescent and treatment 2 bulb also led to higher fruit counts in those plants compared with the rest (21.3 and 21.6 fruits per plant, respectively). Some flowers were unable to develop into fruits, evidenced by the fact that the number of fruits is lower than the number of buds per plant.

Flowering indicators of dragon fruits in the experiment 2 are considerably lower than those in the experiment 1. This is possibly due to the lower temperature in this harvesting season (24.4–24.6°C) compared with the last season (26.9–27.5°C) and is consistent with the experiments of Kim et al. (2009) and of Pin and Nilsson (2012) conducted on facultative long‐day plants. However, in current experiment, CFL bulbs displayed better floral stimulation than incandescent bulbs. To be specific, fruit yields of plants of plants in the treatment 2 and 3 (4.1 and 3.8, respectively) are statistically equal and higher than yields of control and treatment 1 (1.3 and 1.5, respectively).

The experiment 3 was carried out at the same location and under climatic conditions similar to the experiment 1. The experiment results again confirmed the stimulus efficiencies of newly designed light bulbs in the experiment 1 in which plants treated with the incandescent bulbs and CFL bulb 2 showed higher number of floral stems, buds, and fruits per plant compared with those of other plants. Strikingly, plants illuminated with the control and treatment 2 displayed exceptionally large number of bubs per plant (60.4 and 59.4, respectively), approximately 46% higher than plants treated with other treatments and were therefore subjected to bud trimming (to around 25–30 buds/plant) to ensure productivity. Ultimately, trimmed plants showed high fruit yield and were among best white‐flesh dragon fruits plants in terms of yield per plant throughout the three examined harvesting seasons.

Some interesting observations could be made from experiment conducted on red‐flesh dragon fruit plants in Tien Giang province (experiments 4 and 5). Firstly, dragon fruit plants treated with the incandescent light (control) displayed higher number of floral stems, buds, and in turn fruits per plant than those treated with CFL bulbs and even higher than those indicators of the control group in the experiment 3. This suggests that the influence of lamp type on the floral stimulation efficiency is more profound in red‐flesh dragon fruit than in white‐flesh plants and that the development from bud to flowers of the red‐flesh variety is expedited compared with the white‐flesh counterpart under incandescent irradiation. To date, the mechanism explaining for lower supplemented irradiation required for flowering of the red‐flesh dragon fruit has not been clearly elaborated. Nevertheless, current findings on variety‐specific response to artificial irradiation of dragon fruit are in agreement with previous experimental studies asserting that off‐season flowering of red‐flesh dragon fruit could be more easily stimulated than of white‐flesh variety (Be et al., 2014; Yen & Chang, 1997).

Secondly, the CFL treatment 2 performed equally well in comparison with the incandescent light in terms of plant productivity and flowering induction capability in the third harvesting season (experiment 5). To be specific, the number of buds, fruits, and productivity between control and treatment 2 plants displayed no statistical differences. In addition, unlike experiment 4, all plants in this experiment did not require trimming and virtually all buds of dragon fruit plants developed into fruits. This result may carry implication that controlling the number of buds is essential in the light‐induced stimulation process for off‐season fruiting.

Similar to experiment 2, experiment 6 was conducted under colder growing condition (24.9–25.9°C), resulting in comparatively poor growth and productivity. The treatment 2 gave plants with higher number of buds than other groups. In some plants in the control group, no budding was observed. Despite that, productivities and bud numbers of the control and treatment groups in this experiment are still higher than those in the experiment 2, confirming that red‐flesh dragon fruit is more responsive to floral stimulation by artificial lighting. Both thermal and light sensitivity of the flowering process in red‐flesh dragon fruit plant have been confirmed by a previous study where a temperature of at least 15°C was recommended for night lighting for the red‐flesh cultivar (Jiang & Yang, 2015).

Results of experiment 7 generally confirmed the findings of the experiment 5. Both experiments were carried out on the same cultivar and at the same time period. Control incandescent bulb and treatment 2 exerted better floral induction effect on the plant than other lights.

To summarize, the floral stimulation performance of the CFL light bulb of treatment 2 has been shown to be either equal or better than that of the incandescent light bulb through results of field experiments. In six out of seven experiments, treatments 1 and 3 pale in comparison with treatment 2 when it comes to bud‐stimulating capability and productivity. One exception was experiment 4 where treatments 1 and 3 showed comparable floral stimulation capability to that of the treatment 2 and the control bulb was able to result in white‐flesh dragon fruits with marginally better yield per plant than the treatment 2 (15.1 vs. 12.9). Given that the tested CFL lamps have power capacity almost threefold lower than the control, adoption of improved CFL lamps is recommended in energy‐scarce areas with intensive dragon fruit farming activities. In fact, treatment 2 has been commercialized under the code name of CFL‐20 W NN R for flowering stimulation of agricultural crops and ornamental plants in various provinces including Binh Thuan, Long An, Tien Giang, Tay Ninh, Lam Dong, and Ha Noi city since 2016. The product earned good acceptance from farmers due to its affordability and short payback period.

## 4 CONCLUSIONS

In an effort to reduce energy consumption in dragon fruit farming, this study aimed to propose and evaluate new improved CFL lamps specialized for inducing light‐stimulated off‐season flowering in dragon fruit fields. The preliminary survey results indicated that the use of incandescent light bulbs was prevalent among farmers and that domestic CFL lights were generally inefficient in stimulating off‐season flowering in both white‐flesh and red‐flesh dragon fruit cultivars. Emission spectrum measurement of currently used lamps suggested that the intensity in red and far‐red regions of the lamp might be responsible for inducing phytochrome transformation, which is critical in flowering process of the plant. Field experiments adopting three improved CFL light bulbs in three provinces showed that one CFL light (treatment 2) resulted in better flowering in dragon fruit plants than others, which were demonstrated by better growth, blossoming indicators, and productivity. Future studies on combinational strategies in stimulating flowering in dragon fruits are recommended.

## CONFLICT OF INTEREST

The authors declare no conflict of interest.

## Funding information

This study was funded by Ministry of Science and Technology of Vietnam under the project “Design and Manufacture of Specialized Irradiation System and Usage Guidelines for Propagation and Flowering Control of Some Crops at Industrial Scale” (Code: ĐM.06.DN/13) belonging to the National Programme for Technology Innovation to 2020.

## Data Availability

The data that support the findings of this study are available from the corresponding author upon reasonable request.

## APPENDIX A

**TABLE A1:** Plant height, canopy diameter, and the number of stems per plant before and after experiment 1

Indicators	Plant height (cm)		Canopy diameter (cm)		Number of stems per plant	
	Before	After	Before	After	Before	After
Control	160.5	160.7	157.8	156.7	65.6	65.7
Treatment 1	159.8	160.1	157.6	157.8	76.2	76.4
Treatment 2	159.5	160.0	158.3	157.9	72.5	72.6
Treatment 3	158.9	159.7	159.4	159.8	75.5	75.6
CV (%)	1.01	0.15	3.14	0.14	5.72	5.71
LSD 0.05	ns	ns	ns	ns	ns	ns
